# Alternative Polyadenylation of Human Bocavirus at Its 3′ End Is Regulated by Multiple Elements and Affects Capsid Expression

**DOI:** 10.1128/JVI.02026-16

**Published:** 2017-01-18

**Authors:** Sujuan Hao, Junmei Zhang, Zhen Chen, Huanzhou Xu, Hanzhong Wang, Wuxiang Guan

**Affiliations:** a Center for Emerging Infectious Diseases, Wuhan Institute of Virology, Chinese Academy of Sciences, Wuhan, Hubei, China; b University of Chinese Academy of Sciences, Beijing, China; c Hubei Collaborative Innovation Center for Industrial Fermentation, Wuhan, Hubei, China; University of Florida

**Keywords:** HBoV, alternative polyadenylation, capsid protein expression, parvovirus

## Abstract

Alternative processing of human bocavirus (HBoV) P5 promoter-transcribed RNA is critical for generating the structural and nonstructural protein-encoding mRNA transcripts. The regulatory mechanism by which HBoV RNA transcripts are polyadenylated at proximal [(pA)p] or distal [(pA)d] polyadenylation sites is still unclear. We constructed a recombinant HBoV infectious clone to study the alternative polyadenylation regulation of HBoV. Surprisingly, in addition to the reported distal polyadenylation site, (pA)d, a novel distal polyadenylation site, (pA)d2, which is located in the right-end hairpin (REH), was identified during infectious clone transfection or recombinant virus infection. (pA)d2 does not contain typical hexanucleotide polyadenylation signal, upstream elements (USE), or downstream elements (DSE) according to sequence analysis. Further study showed that HBoV nonstructural protein NS1, REH, and *cis* elements of (pA)d were necessary and sufficient for efficient polyadenylation at (pA)d2. The distance and sequences between (pA)d and (pA)d2 also played a key role in the regulation of polyadenylation at (pA)d2. Finally, we demonstrated that efficient polyadenylation at (pA)d2 resulted in increased HBoV capsid mRNA transcripts and protein translation. Thus, our study revealed that all the bocaviruses have distal poly(A) signals on the right-end palindromic terminus, and alternative polyadenylation at the HBoV 3′ end regulates its capsid expression.

**IMPORTANCE** The distal polyadenylation site, (pA)d, of HBoV is located about 400 nucleotides (nt) from the right-end palindromic terminus, which is different from those of bovine parvovirus (BPV) and canine minute virus (MVC) in the same genus whose distal polyadenylation is located in the right-end stem-loop structure. A novel polyadenylation site, (pA)d2, was identified in the right-end hairpin of HBoV during infectious clone transfection or recombinant virus infection. Sequence analysis showed that (pA)d2 does not contain typical polyadenylation signals, and the last 42 nt form a stem-loop structure which is almost identical to that of MVC. Further study showed that NS1, REH, and *cis* elements of (pA)d are required for efficient polyadenylation at (pA)d2. Polyadenylation at (pA)d2 enhances capsid expression. Our study demonstrates alternative polyadenylation at the 3′ end of HBoV and suggests an additional mechanism by which capsid expression is regulated.

## INTRODUCTION

Human bocavirus (HBoV) was first identified in 2005 from pooled nasopharyngeal aspirates from patients with respiratory diseases (1). HBoV is classified in the genus Bocaparvovirus within the family Parvoviridae (2). Different HBoV species, including the prototype HBoV, originally isolated from respiratory samples, as well as HBoV2, HBoV3, and HBoV4, isolated from feces, are commonly detected in infants (3–12). HBoV is associated with lower-respiratory-tract infections and gastroenteritis (3, 8, 13, 14) and, in many cases, coinfections with other viruses (15–20).

The HBoV genome is a 5.5-kb single-stranded DNA, with hairpins at both ends which are necessary for viral genome replication (21, 22). The left-end hairpin contains a bubble region and an asymmetric T, while the right end harbors a stem-loop structure (21). mRNA transcripts are generated from alternatively processed mRNA precursor transcribed from the promoter located at map unit 5 (P5). The left half of the HBoV genome encodes the nonstructural protein NS1, which is essential for viral replication. Like other parvoviruses, HBoV NS1 contains DNA-binding and endonuclease domains in the N terminus, ATPase and helicase domains in the center, and a transactivation domain at the C terminus (23). The nonstructural protein NP1 open reading frame (ORF) is located in the middle of the viral genome. NP1 is required for the efficient replication of viral DNA, facilitates VP-encoding mRNA transcripts to read through the internal polyadenylation site, and plays a role in controlling the production of VP mRNAs (21, 24, 25). The right half of the genome encodes the structural proteins VP1 and VP2, which share a C terminus, while the N terminus of VP1 contains a phospholipase A2 (PLA2) domain which is essential for parvovirus infectivity (26, 27).

Similar to bovine parvovirus (BPV) and minute virus of canines (MVC), HBoV contains multiple proximal polyadenylation sites (22, 28). Blockage to the production of full-length capsid-encoding transcripts by (pA)p in the middle of the viral genome could be a limiting step for parvovirus infection (29, 30). All of the bocaviruses contain a similar stem-loop structure at the right end. The distal polyadenylation sites, (pA)d, of BPV and MVC are located on the right-end palindromic terminus (31, 32), and the capsid mRNA transcripts of BPV and MVC are polyadenylated in the loop of stem-loop structures (32). However, the distal polyadenylation site of HBoV is located about 400 nucleotides (nt) upstream of the right-end hairpin and is polyadenylated downstream of the hexanucleotide AAUAAA. The differences among the (pA)d sites in BPV, MVC, and HBoV raise the question of how HBoV polyadenylation at (pA)d is regulated.

Here, we constructed a recombinant HBoV infectious clone and investigated the regulation of polyadenylation at (pA)d either during recombinant HBoV infection or after infectious clone transfection. We discovered a new polyadenylation site, (pA)d2, in the right-end palindromic terminus, which is similar to those of BPV and MVC. Further study showed polyadenylation at (pA)d2 is regulated by nonstructural protein NS1, right-end hairpin (REH), and *cis* elements of (pA)d. Importantly, the regulation of polyadenylation at (pA)d2 is linked to capsid protein expression levels, which could be important for virus production.

## RESULTS

### A distal polyadenylation site is located in the right-end hairpin.

**FIG 1:**
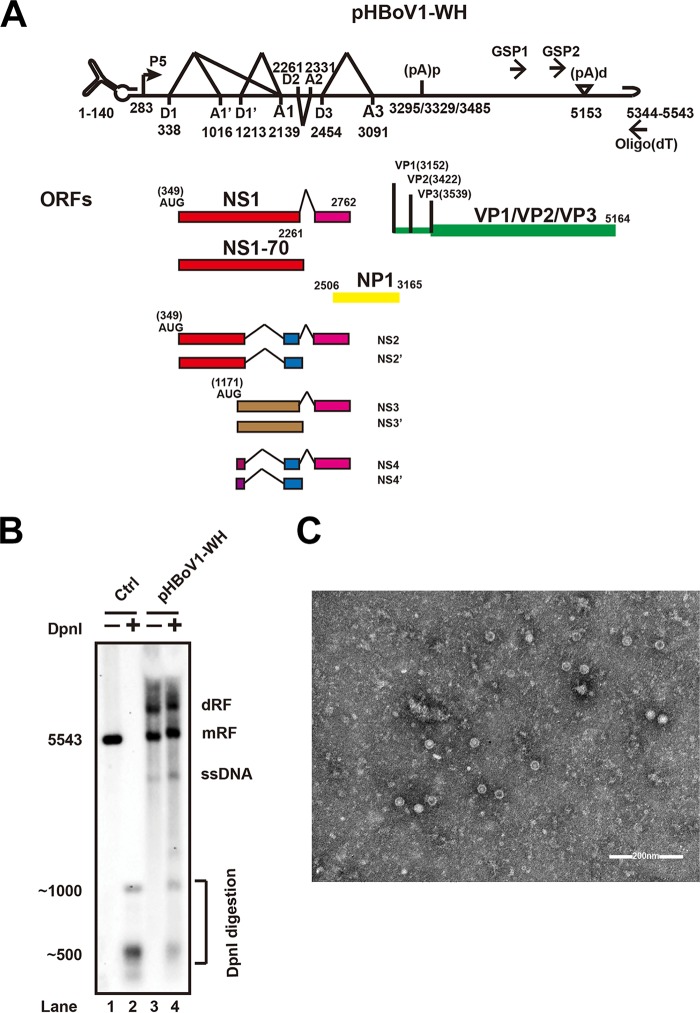
Infectious clone construction and recombinant virus production. (A) Transcription map of HBoV. Genetic map of HBoV is depicted, highlighting the P5 promoter, splicing donor sites (D), splicing acceptor sites (A), proximal polyadenylation site [(pA)p], and distal polyadenylation site [(pA)d]. The open reading frames are diagrammed. The primers for 3′ RACE are indicated by GSP1, GSP2, and oligo(dT). The numbers denote the nucleotide positions in the HBoV genome. (B) Southern blot analysis. Hirt DNAs were isolated from recombinant infectious HBoV clone-transfected HEK293T cells. DpnI-digested fragments were resolved in 1% agarose gels and transferred to Hybond-N^+^ membrane, followed by hybridization of an HBoV probe spanning from nt 1 to nt 5543. Lane 2 shows the size of bands after digesting the HBoV genomic DNA with DpnI. dRF, double replication form; mRF, monomer replication form. (C) Purified recombinant HBoV viral particles were negatively stained and analyzed using transmission electron microscopy.

The HBoV (Wuhan isolate) nonstructural (NS1) and capsid (VP1/VP2) protein-encoding genes were amplified from a DNA extract prepared from the feces of a 2-year-old infant. Synthesized left-end hairpin (LEH) and REH sequences, together with NS- and capsid-encoding genes, were ligated into the pBBSmal vector (21). The full-length recombinant clone was designated pHBoV1-WH (Fig. 1A) and transfected into HEK293T cells. DpnI-resistant bands and single-stranded DNA (ssDNA) were detected (Fig. 1B, lane 4), which suggested that pHBoV1-WH replicated in HEK293T cells. Large-scale pHBoV1-WH transfection and gradient iodixanol centrifugation were performed to purify the progeny virion for electron microscopy analysis. A typical icosahedral structure of the purified virus is shown (Fig. 1C).

**FIG 2:**
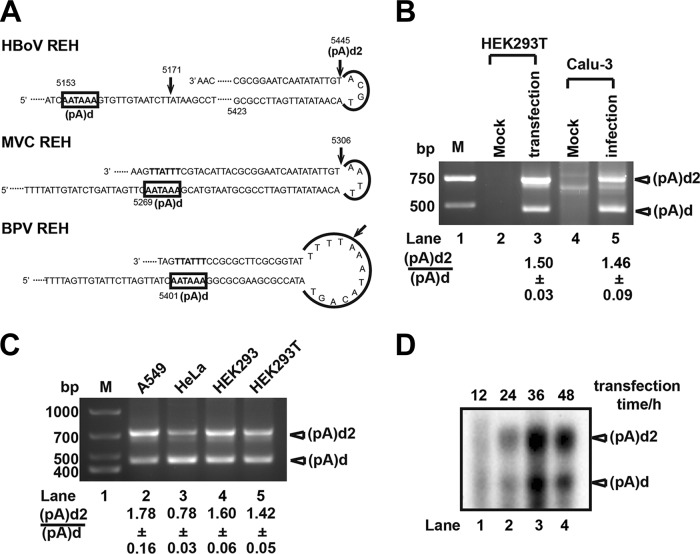
Discovery of distal polyadenylation site (pA)d2. (A) Polyadenylation sites of different bocaparvoviruses located in the REH. (B) 3′ RACE. Total RNA was isolated from recombinant virus-infected Calu-3 cells or pHBoV1-WH-transfected HEK293T cells and reverse transcribed with oligo(dT). PCR was performed with specific primers and products were resolved in 1.5% agarose gels. (C) 3′ RACE. pHBoV1-WH was transfected to A549, HeLa, HEK293, or HEK293T cells, RNA was isolated, and 3′ RACE was performed as described for panel B. Lane M, DNA ladder. (D) Northern blot analysis. Total RNA was isolated from HBoV infectious clone-transfected HEK293T cells at the indicated times and hybridized with oligo(dT) and HBoV-specific primers, followed by RNase H cleavage. RNAs were resolved in 1.5% agarose gels, transferred to Hybond-N^+^ membranes, and hybridized with probes spanning nt 4745 to nt 5171.

To investigate the 3′ terminal sequences of the viral mRNA transcripts, pHBoV1-WH was transfected into HEK293T cells, and 3′ rapid amplification of cDNA ends (RACE) was performed with primers specific for the proximal and distal polyadenylation sites. Consistent with previous reports (21, 22, 28), sequence analysis of 3′ RACE data showed that the nonstructural protein-coding genes (NS1 and NP1) were polyadenylated at proximal sites (nt 3295, nt 3329, and nt 3485) (Fig. 1A). Polyadenylation at distal sites were detected at nt 5171 [(pA)d] and nt 5445 [(pA)d2] when pHBoV1-WH was transfected into different cell lines (Fig. 2C, lanes 2 to 5) or when polarized Calu-3 cells were infected with purified virus (Fig. 2B, lane 5).

The distal polyadenylation site (pA)d has a typical polyadenylation signal hexanucleotide, AAUAAA, cleavage site, and potential downstream elements. However, sequence analysis showed (pA)d2 does not contain these key elements. Similar to the distal polyadenylation of BPV and MVC, (pA)d2 is located on the right-end terminus (Fig. 2A). Interestingly, the last 42 nt of the right-end stem of HBoV and MVC are almost identical, with only two different nucleotides in the loop (Fig. 2A). To confirm our 3′ RACE results, total RNA of pHBoV1-WH-transfected HEK293T cells were harvested at different times (12, 24, 36, and 48 h) and hybridized with oligo(dT) and specific primers upstream of (pA)d. RNA fragments were detected with probes spanning from nt 4745 to nt 5171 after RNase H cleavage. Polyadenylation at (pA)d and (pA)d2 were both detected by Northern blot analysis (Fig. 2D), which is consistent with 3′ RACE data and suggests that a portion of VP1/VP2 encoding mRNA transcripts are polyadenylated at (pA)d2.

### NS1 and REH are required for polyadenylation at (pA)d2.

**FIG 3:**
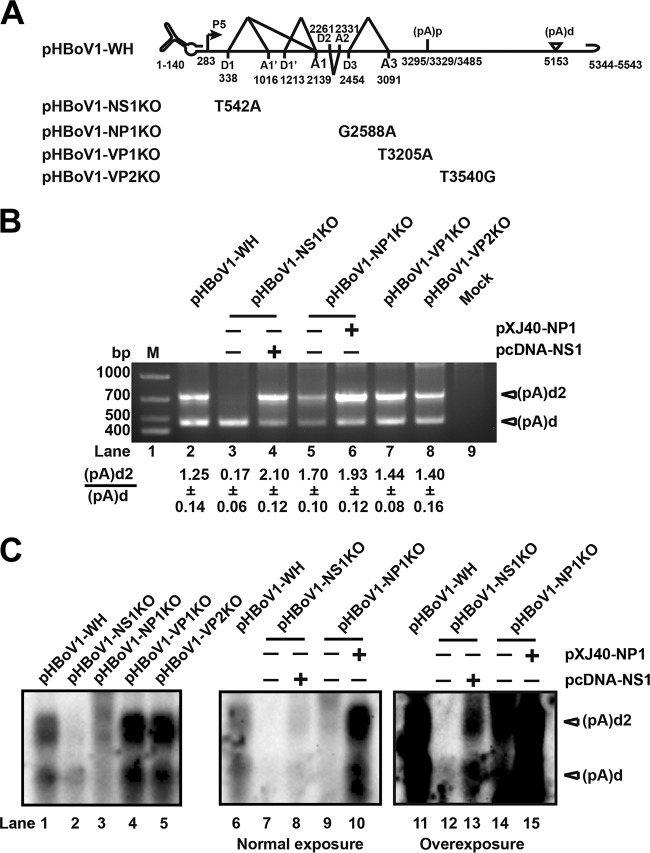
NS1 is required for efficient polyadenylation at (pA)d2. (A) Infectious clone pHBoV1-WH and mutants are shown. (B) 3′ RACE. Total RNA was isolated from HEK293T cells transfected with the plasmids shown in panel A, and then 3′ RACE was performed as described in the legend to Fig. 2B. (C) Northern blot analysis. Total RNA was isolated from HEK293T cells transfected with the plasmids shown in panel A, and then RNase H cleavage and Northern blotting were performed as described in the legend to Fig. 2D. The image in the middle (lanes 6 to 10) and the image on the right (lanes 11 to 15) were of the same batch of samples with different exposure times.

Since (pA)d2 does not contain a typical polyadenylation signal, we determined whether the viral proteins affect polyadenylation at (pA)d2. All of the structural and nonstructural protein open reading frames were disrupted by single-nucleotide mutation, which resulted in early translation termination (Fig. 3A). Transfection of the NS1 knockout plasmid (pHBoV1-NS1KO) resulted in a 7-fold reduction in polyadenylation at (pA)d2 (Fig. 3B, lane 3, and C, lane 2). Cotransfection of pHBoV1-NS1KO and NS1 expression plasmid (pcDNA-NS1) restored polyadenylation at (pA)d2 (Fig. 3B, lane 4, and C, lanes 8 and 13), which suggested that NS1 is necessary for efficient polyadenylation at (pA)d2. Transfection of the NP1 knockout plasmid (pHBoV1-NP1KO) to HEK293T cells resulted in decreased polyadenylation at both (pA)d and (pA)d2 as measured by using 3′ RACE (Fig. 3B, lanes 5) and Northern blot analysis (Fig. 3C, lanes 3 and 9). However, the ratio of polyadenylation at (pA)d2 to (pA)d was not changed. Cotransfection of pHBoV1-NP1KO and NP1 expression plasmid (pXJ40-NP1) did not change the ratio of polyadenylation at (pA)d2 to (pA)d (Fig. 3B, lane 6, and C, lane 10). However, the abundance of RNA transcripts polyadenylated at both (pA)d and (pA)d2 (Fig. 3B, lane 6, and C, lane 10) increased more than 10-fold, which is consistent with the previous report that NP1 facilitates capsid protein expression (33). These results suggested that NP1 is not required for polyadenylation at (pA)d2.

**FIG 4:**
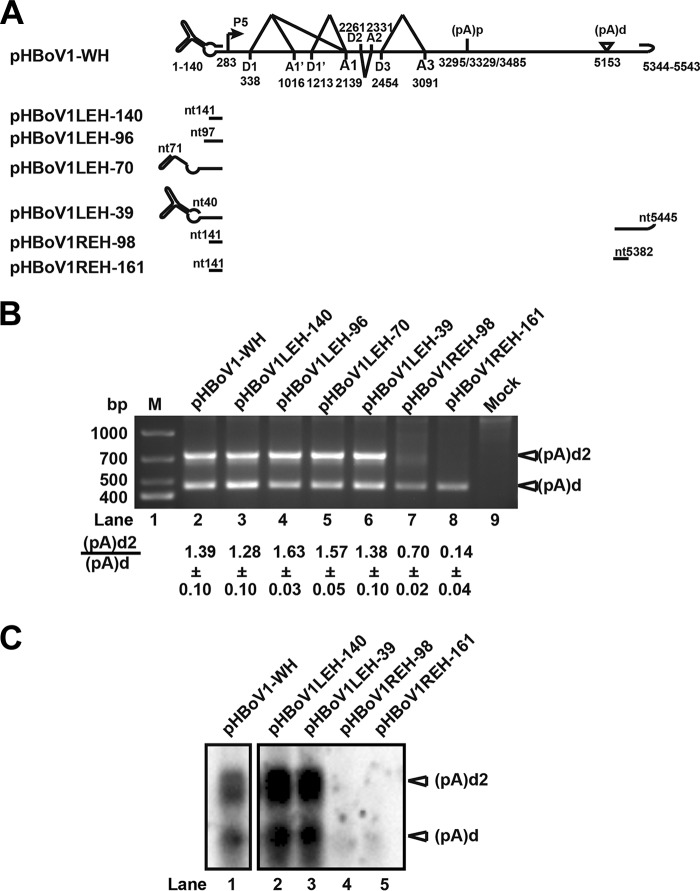
REH is required for polyadenylation at (pA)d2. (A) LEH and REH mutants of pHBoV1-WH are shown. (B) 3′ RACE. Total RNA was isolated from HEK293T cells transfected with the plasmids shown in panel A, and then 3′ RACE was performed as described in the legend to Fig. 2B. (C) Northern blot analysis. Total RNA isolated from transfected HEK293T cells was resolved in 1.5% agarose gels, transferred to Hybond-N^+^ membranes, and hybridized with probes spanning nt 4745 to nt 5171.

Parvovirus VP1 is required for viral infection, and VP2 is the major capsid protein which is involved in viral particle assembly. Transfecting VP1 and VP2 knockout plasmids (pHBoV1-VP1KO and pHBoV1-VP2KO) to HEK293T cells did not result in a change of polyadenylation at (pA)d2 (Fig. 3B, lanes 7 and 8, and C, lanes 4 and 5), which suggested that VP1/VP2 is not involved in the regulation of its 3′-end alternative polyadenylation.

(pA)d2 is located in the loop of the right-end palindromic terminus. We next checked whether the hairpins at 5′ and/or 3′ ends of HBoV are necessary for polyadenylation at (pA)d2. Even deleting all of the left-end hairpin did not affect polyadenylation at (pA)d2 (Fig. 4B, lanes 3 to 6, and C, lanes 2 and 3). Transfecting pHBoV1REH-98, in which the sequences after the (pA)d2 site (nt 5445) were truncated, to HEK293T cells resulted in decreased polyadenylation at (pA)d2, and the (pA)d2/(pA)d ratio was reduced 2-fold (Fig. 4B, lane 7). Polyadenylation only at (pA)d was observed when pHBoVREH-161, in which the sequences before (pA)d2 were trimmed, was transfected to HEK293T cells (Fig. 4B, lane 8). The result indicated that the right hairpin plays an important role in polyadenylation at (pA)d2.

Taken together, these data show that NS1 and REH are required for polyadenylation at (pA)d2, while NP1 and capsid proteins do not regulate polyadenylation at (pA)d2.

### *cis* elements of (pA)d are required for polyadenylation at (pA)d2.

**FIG 5:**
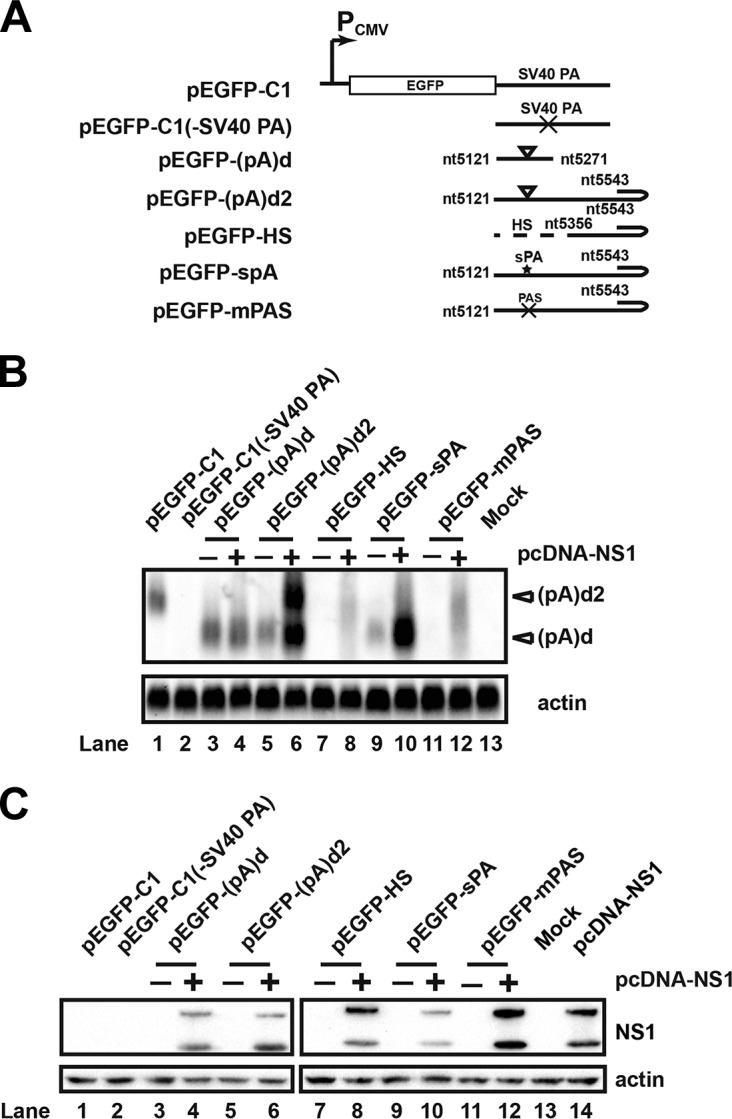
NS1, REH, and *cis* elements of (pA)d are required for polyadenylation at (pA)d2. (A) Diagram of (pA)d reporter plasmids. (pA)d and downstream sequence were inserted into pEGFP-C1 to replace the SV40 polyadenylation signal. (B) Northern blot analysis. Total RNA was isolated from transfected HEK293T cells, and Northern blotting was performed as described in the legend to Fig. 2D. (C) Western blot. HEK293T cells were transfected with an HA-tagged NS1 expression plasmid and analyzed using Western blotting 48 h posttransfection.

To test further which elements are required for efficient polyadenylation at (pA)d2, we constructed an alternative polyadenylation reporter system, in which (pA)d and downstream sequences were cloned into pEGFP-C1 vector to replace the simian virus 40 (SV40) polyadenylation signal (Fig. 5A). Transfection of plasmid pEGFP-(pA)d, which contains (pA)d and downstream sequences but no REH sequences, into HEK293T cells resulted in polyadenylation only at (pA)d in the absence or presence of NS1 cotransfection (Fig. 5B, lanes 3 and 4, and C, lanes 3 and 4). Transfecting plasmid pEGFP-(pA)d2, which contains (pA)d and REH sequences, into HEK293T cells resulted in polyadenylation at (pA)d in the absence of NS1 cotransfection (Fig. 5B, lane 5, and C, lane 5). However, cotransfection of pEGFP-(pA)d2 with pcDNA-NS1 (Fig. 5C, lane 6) to HEK293T cells resulted in polyadenylation at both (pA)d and (pA)d2, and the level of RNA transcripts increased more than 10-fold (Fig. 5B, lane 6). These results are consistent with the previous results, indicating that both NS1 and REH are required for efficient polyadenylation at (pA)d2.

To investigate whether the *cis* elements of (pA)d are required for polyadenylation at (pA)d2, (pA)d and downstream sequences were replaced with heterologous sequences, as diagrammed in Fig. 5A. Transfecting pEGFP-HS (Fig. 5B, lanes 7 and 8) into HEK293T cells resulted in a loss of polyadenylation at (pA)d in the presence of NS1 cotransfection (Fig. 5C, lane 8), which suggested that *cis* elements of (pA)d are required for polyadenylation at (pA)d2. We then mutated the hexanucleotide of the (pA)d (pEGFP-mPAS). No polyadenylation at (pA)d2 was observed when pEGFP-mPAS was transfected into cells in the absence or presence of NS1 cotransfection (Fig. 5B, lanes 11 and 12, and C, lanes 11 and 12). Replacing (pA)d and downstream sequences with a synthetic polyadenylation signal (sPA) resulted in strong polyadenylation at sPA without polyadenylation at (pA)d2, even in the presence of NS1 cotransfection (Fig. 5B, lanes 9 and 10, and C, lanes 9 and 10). Those results indicated that the addition of the sPA created a strong enough polyadenylation signal that the majority of RNA transcripts were polyadenylated at (pA)d, and few RNA transcripts were polyadenylated at (pA)d2.

Collectively, these experiments showed that NS1, REH, and *cis* elements of (pA)d are sufficient viral elements for efficient polyadenylation at (pA)d2.

### DSE of (pA)d regulate alternative polyadenylation at (pA)d and (pA)d2.

**FIG 6:**
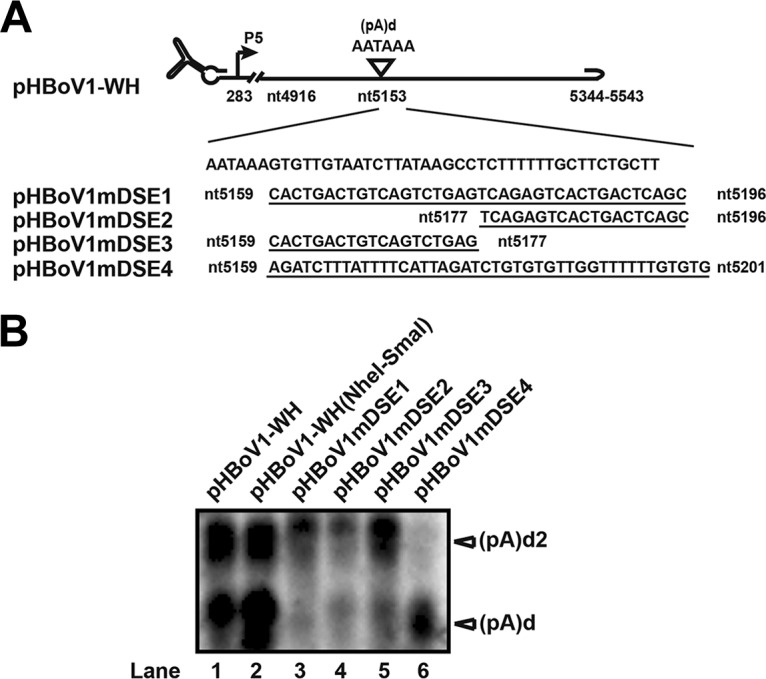
Downstream elements (DSE) of (pA)d regulate polyadenylation at (pA)d and (pA)d2. (A) Diagram of (pA)d and its mutant plasmids. Downstream sequences of (pA)d were mutated as diagrammed. (B) Northern blot analysis. Total RNA was isolated from transfected HEK293T cells. RNase H cleavage and Northern blotting were performed as described in the legend to Fig. 2D.

We then determined whether downstream elements (DSE) of (pA)d affect polyadenylation at (pA)d2 in the context of an infectious clone. Sequence analysis showed that a U-rich stretch is 22 nt downstream of hexanucleotide AAUAAA. Transfecting the U-rich mutation plasmids (pHBoV1mDSE1 and pHBoV1mDSE2) into HEK293T cells resulted in reduced polyadenylation at both (pA)d and (pA)d2, as determined by using Northern blotting (Fig. 6B, lanes 3 and 4), suggesting that this motif is a major component of downstream elements for efficient polyadenylation at (pA)d and (pA)d2. Mutating the sequences between the U-stretch and AAUAAA resulted in reduced polyadenylation at (pA)d, but polyadenylation at (pA)d2 was not changed (Fig. 6B, lane 5). This result shows that polyadenylation at (pA)d is not required for polyadenylation at (pA)d2. There is also a difference in the requirement of *cis* elements for polyadenylation at the two different sites. The sequences between nt 5159 and nt 5196 are required for efficient polyadenylation at (pA)d. Polyadenylation at (pA)d2 only requires sequences between nt 5178 and nt 5196. Replacing the (pA)d and downstream sequences with a synthetic polyadenylation signal resulted in strong polyadenylation at (pA)d but a loss of polyadenylation at (pA)d2 (Fig. 6B, lane 6), which further confirmed that a strong polyadenylation at (pA)d inhibited polyadenylation at (pA)d2.

### The sequences and distance between (pA)d and (pA)d2 affect polyadenylation at (pA)d2.

**FIG 7:**
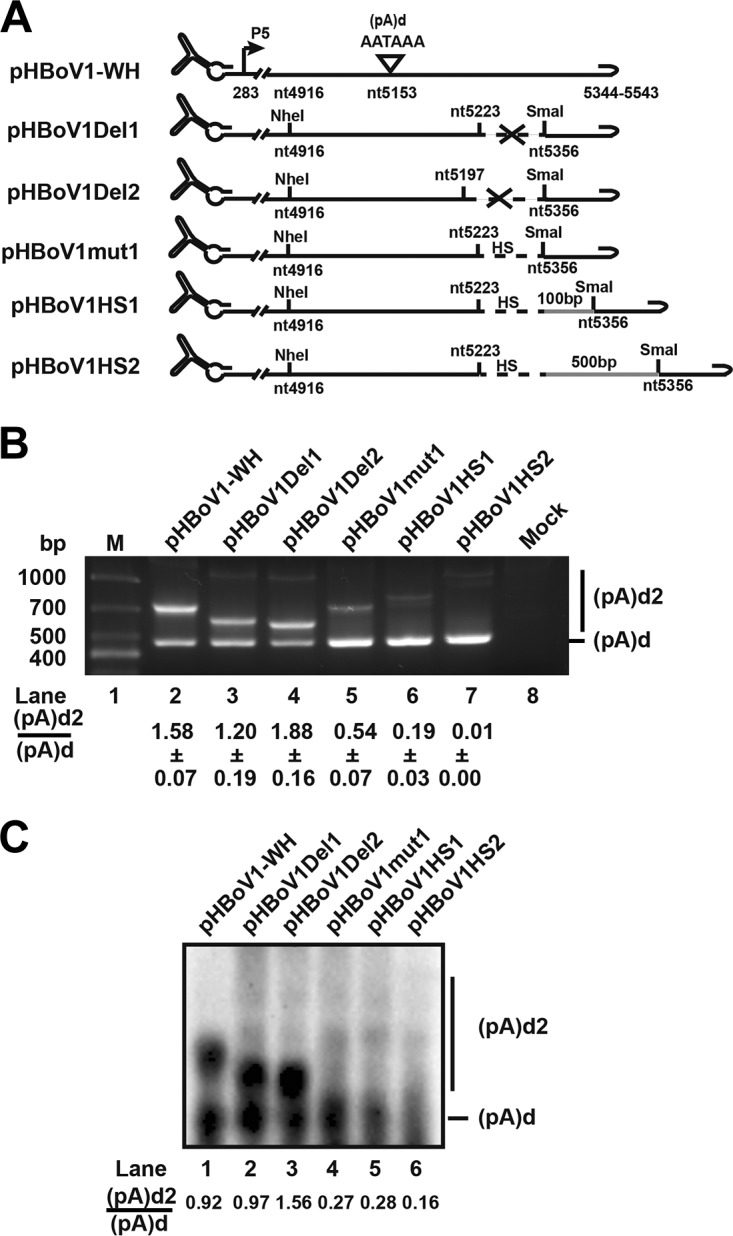
Sequence and distance between (pA)d and (pA)d2 affect polyadenylation at (pA)d2. (A) Diagram of pHBoV1-WH and its mutants. The sequence between nt 5223 or nt 5197 and nt 5356 was deleted in pHBoV1Del1 and pHBoV1Del2, respectively. In plasmids pHBoV1mut1, pHBoV1HS1, and pHBoV1HS2, the sequence between nt 5223 and nt 5356 was replaced with heterologous sequence, as indicated. (B) 3′ RACE was performed as described in the legend to Fig. 2B. (C) Northern blotting was performed as described in the legend to Fig. 2D.

We next investigated whether the distance between (pA)d and (pA)d2 plays a role in the regulation of alternative polyadenylation at the 3′ end of HBoV. pHBoV1Del1 and pHBoV1Del2, in which the sequences from nt 5223 or nt 5197 to nt 5356 were deleted, were transfected into HEK293T cells. 3′ RACE and Northern blot analysis showed that neither polyadenylation at (pA)d2 nor the (pA)d2/(pA)d ratio changed (Fig. 7B, lanes 3 and 4, and C, lanes 2 and 3), which indicates shortening the distance does not affect polyadenylation at (pA)d2. As the distance between the two polyadenylation sites was increased by the addition of heterologous sequences, polyadenylation at (pA)d2 decreased (Fig. 7B, lanes 6 and 7, and C, lanes 5 and 6), and the (pA)d2/(pA)d ratio decreased more than 8-fold. Replacing the sequence between (pA)d and (pA)d2 with heterologous sequences also resulted in decreased polyadenylation at (pA)d2 (Fig. 7B, lane 5, and C, lane 4), which implicated that the sequences between (pA)d and (pA)d2 are also important for alternative polyadenylation regulation.

### Polyadenylation at (pA)d2 enhances capsid mRNA and protein expression.

**FIG 8:**
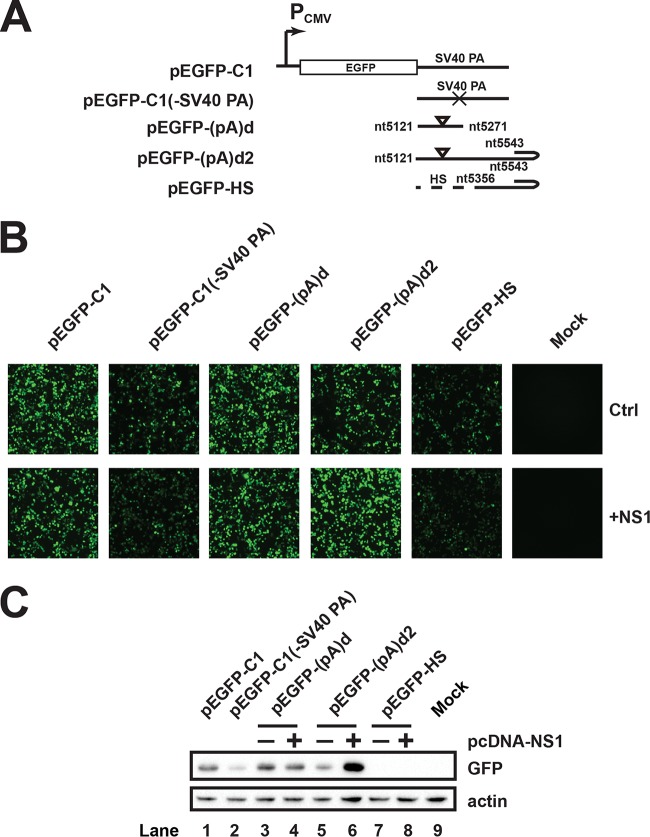
Alternative polyadenylation at (pA)d2 increases the expression level of GFP protein. (A) Diagram of (pA)d2 reporter plasmids. (B) Microscopy detection of GFP expression. (C) Western blotting was performed as described in the legend to Fig. 5C using anti-GFP antibody.

The outcome of alternative polyadenylation is to regulate the expression level of encoded proteins. To determine whether polyadenylation at (pA)d2 affects protein expression, we first measured whether (pA)d2 affects green fluorescent protein (GFP) expression in the reporter system. GFP expression did not change obviously (Fig. 8B and C, lanes 3 and 4) when pEGFP-(pA)d was transfected into HEK293T cells in the presence of NS1 cotransfection. However, in the presence of NS1 expression, transfection of pEGFP-(pA)d2 resulted in polyadenylation at both (pA)d and (pA)d2, and the expression of GFP increased as determined using immunofluorescence and Western blot analysis (Fig. 8B and C, lanes 5 and 6). Transfecting pEGFP-HS resulted in the loss of polyadenylation at (pA)d2 and a decrease in GFP expression (Fig. 8B and C, lanes 7 and 8), suggesting that polyadenylation at (pA)d2 regulates mRNA and protein expression levels.

**FIG 9:**
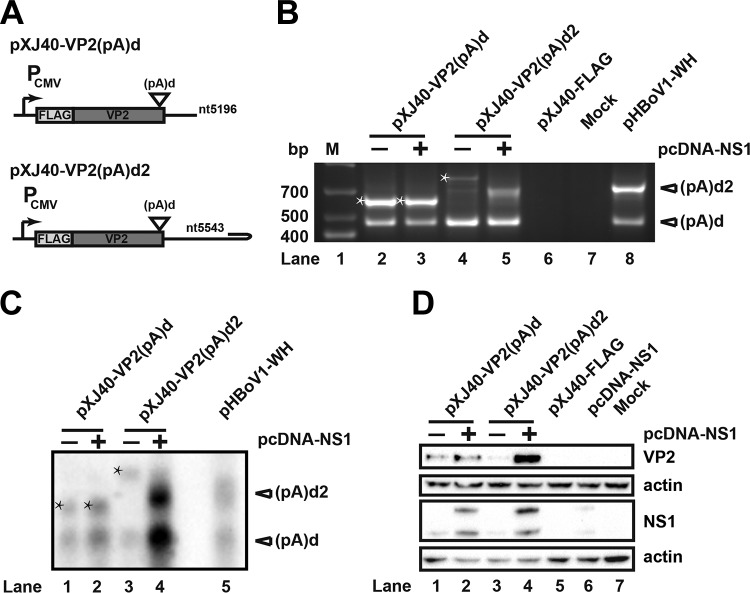
Alternative polyadenylation at (pA)d2 increases expression level of capsid protein. (A) VP2 expression plasmids with or without (pA)d2 are shown. (B) 3′ RACE was performed as described in the legend to Fig. 2B. Stars represent RNAs that were polyadenylated at the vector polyadenylation site. (C) Northern blot analysis was performed as described in the legend to Fig. 2D. Bands with stars represent RNAs that were polyadenylated at the vector polyadenylation site. (D) Western blot. HEK293T cells were transfected with HA-tagged NS1 or capsid expression plasmids. The transfected cells were lysed for Western blot analysis with HA antibody, FLAG antibody, and β-actin.

To analyze whether polyadenylation affects capsid protein expression, a VP2 expression cassette and the downstream sequence of (pA)d, with or without (pA)d2, were cloned into expression vector pXJ40-FLAG. Cotransfecting pXJ40-VP2(pA)d and an NS1 expression plasmid into HEK293T cells resulted in polyadenylation at (pA)d and the polyadenylation site of pXJ40-FLAG vector (Fig. 9B, lanes 2 and 3). No polyadenylation at (pA)d2 was detected because of the lack of REH (Fig. 9B, lanes 2 and 3, and C, lanes 1 and 2). Similar levels of VP2 mRNA and protein were observed (Fig. 9C, lanes 1 and 2, and D, lanes 1 and 2). However, cotransfecting pXJ40-VP2(pA)d2 with NS1 into HEK293T cells resulted in polyadenylation at both (pA)d and (pA)d2 (Fig. 9B, lane 5, and C, lane 4). The level of mRNA transcripts increased more than 20-fold when NS1 was cotransfected with pXJ40-VP2 (pA)d2 (Fig. 9C, lanes 3 and 4). The expression level of VP2 protein increased at least 10-fold (Fig. 9D, lanes 3 and 4), suggesting that polyadenylation at (pA)d2 increased the mRNA and protein expression level of capsid. Taken together, these data suggest that alternative polyadenylation at the 3′ end of HBoV regulates the capsid mRNA transcript levels and results in increased capsid protein expression.

## DISCUSSION

We discovered a new polyadenylation site, (pA)d2, in the right-end hairpin of human bocavirus. The nonstructural protein NS1, the right-end hairpin, and *cis* elements of (pA)d are all required for efficient polyadenylation at (pA)d2. The sequences and distance between (pA)d and (pA)d2 also affect polyadenylation efficiency at (pA)d2. Increased polyadenylation at (pA)d2 resulted in elevated capsid expression, indicating that alternative polyadenylation at the 3′ end of HBoV is important for virus structural protein expression.

### The discovery of new distal polyadenylation site (pA)d2.

Alternative polyadenylation plays an important role in the parvovirus life cycle. The blockage of RNA transcript maturation by the proximal polyadenylation site is a limiting step of parvovirus infection (29, 30, 34, 35). When B19V infects permissive cells, most RNA transcripts read through proximal polyadenylation sites, and full-length mRNA transcripts are generated to encode capsid proteins for the production of progeny virus. However, when B19V infects nonpermissive cells, the majority of RNA transcripts are polyadenylated at (pA)p, causing a lack of capsid mRNA, resulting in aborted infection. Internal polyadenylation is also a limiting step of Aleutian mink disease virus (AMDV) genome replication and progeny virus production (30).

We found that the recombinant HBoV infectious clone contains three proximal polyadenylation sites, which is consistent with previous reports (22, 28). Interestingly, in addition to the reported distal polyadenylation site, (pA)d, a new polyadenylation site, (pA)d2, was discovered when recombinant infectious clones were transfected into HEK293T cells. In contrast to the distal polyadenylation sites of BPV and MVC, which are located in the right-end palindromic terminus, the (pA)d of HBoV is located about 400 nt upstream of the right-end hairpin. VP1/VP2-encoding mRNAs are efficiently polyadenylated downstream of the AAUAAA site. Polyadenylation signals include the hexanucleotide AAUAAA or its variants, the cleavage site CA dinucleotide, downstream U-rich elements, and G/U-rich upstream elements. However, (pA)d2 of HBoV is located in the loop of REH, and no classical hexanucleotide polyadenylation signal was found around (pA)d2. The last 42 nucleotides of the stem-loop structure of HBoV and MVC are almost identical. It is possible that HBoV and MVC use similar mechanisms for polyadenylation at the stem-loop region. The only puzzling thing is that there is no polyadenylation site immediately upstream of (pA)d2. To our knowledge, this is the first report of alternative polyadenylation at a distal polyadenylation site in parvoviruses.

### Regulation of polyadenylation at (pA)d2 by *cis* and *trans* elements.

HBoV encodes at least three nonstructural proteins that include NS1, NS1-70K, and NP1. NS1 is a multifunctional protein expressed from the mRNA transcripts from the P5 promoter of the left half of the genome. NS1 contains DNA binding, endonuclease, ATPase, and helicase activities. Disrupting the NS1 open reading frame by point mutation resulted in the loss of polyadenylation at (pA)d2, suggesting that NS1 is indispensable for polyadenylation at (pA)d2. NS2, NS3, and NS4 are dispensable for viral replication (36). However, NS2 is necessary for efficient replication in primary human airway epithelium cultured at an air-liquid interface (HAE-ALI). Polyadenylation at (pA)d2 was not detected after cotransfection of NS2, NS3, NS4, and NS1-70K expression plasmids with (pA)d2 polyadenylation reporter plasmid pEGFP-(pA)d2 in HEK293T cells (data not shown). These results suggested that all of the functional NS1 domains are required for efficient polyadenylation at (pA)d2.

NP1 is unique among parvoviruses and is essential for viral genome DNA replication (21, 32). NP1 facilitates (pA)p read-through of structural protein-encoding mRNA transcripts (24, 25) and increases capsid protein expression. Disrupting the NP1 open reading frame did not affect polyadenylation at (pA)d and (pA)d2, while the RNA transcript abundance decreased. Cotransfection of NP1-expressing plasmid pXJ40-NP1 with pHBoV1-NP1KO did not change the ratio of polyadenylation at (pA)d2 to (pA)d. However, the total RNA level increased, which indicates NP1 regulates the abundance of mRNA transcripts but not polyadenylation at (pA)d2. Structural proteins VP1/VP2 have no effect on polyadenylation at (pA)d2.

Since no typical polyadenylation sequence was found before the (pA)d2 site, whether the *cis* elements of (pA)d are important for efficient polyadenylation at (pA)d2 was determined by using mutagenesis. Mutating either the hexanucleotide AAUAAA or the DSE resulted in decreased polyadenylation at both (pA)d and (pA)d2, indicating that AAUAAA and DSE of (pA)d are indispensable for the efficient polyadenylation at (pA)d2. Interestingly, we found that polyadenylation at (pA)d2 was detected when polyadenylation at (pA)d was abolished by mutation of sequences from nt 5159 to nt 5177, which suggested that polyadenylation at (pA)d is not required for polyadenylation at (pA)d2. The downstream elements required for polyadenylation at (pA)d and (pA)d2 are also different. The distance and sequence *per se* between (pA)d and (pA)d2 also affected polyadenylation efficiency at (pA)d2. Polyadenylation at (pA)d2 was decreased when the distance was increased or the sequence was replaced with heterologous sequence. Thus, polyadenylation at (pA)d2 depends on the *cis* elements of (pA)d. Mutating the right-end hairpin structure destroyed the stem-loop structure where (pA)d2 is located and resulted in the loss of polyadenylation at (pA)d2, indicating that the hairpin structure also plays an important role in polyadenylation at (pA)d2.

### (pA)d2 function.

Alternative polyadenylation is widely spread in metazoan protein-coding transcripts and leads to variable 3′ untranslated regions (UTRs), which has been shown to regulate gene expression (37, 38). In the presence of the NS1 expression plasmid, transfecting pXJ40-VP2(pA)d2 resulted in polyadenylation at (pA)d and (pA)d2. We found that polyadenylation at (pA)d2 increased not only the abundance of VP1/VP2 transcripts but also the expression level of VP1/VP2 proteins. However, our results do not immediately suggest a mechanism to explain the increase in capsid protein expression, a topic we are currently investigating. The distance between (pA)d and (pA)d2 is 274 nt and affects polyadenylation efficiency at (pA)d2. This 3′ noncoding region (NCR) of HBoV has been predicted to form two conserved hairpin structures and plays an important part in the replication of bocaviruses (39–41). The 3′ NCR could function as a binding site for miRNAs or be involved in the production of noncoding RNAs to regulate protein expression (42, 43). The sequences of this region enhanced polyadenylation at (pA)d2, but the mechanism is currently unknown. Taken together, our study has demonstrated that alternative polyadenylation of HBoV capsid-encoding mRNA transcripts is regulated both by the nonstructural protein NS1 and by multiple *cis* elements.

## MATERIALS AND METHODS

### Cell lines and cell culture.

HEK293T (ATCC, CRL-11268), HEK293 (ATCC, CRL-1573), A549 (ATCC, CCL-185), and HeLa cells (ATCC, CCL-2) were cultured in Dulbecco's modified Eagle's medium (DMEM; GIBCO) containing 10% fetal bovine serum (FBS; GIBCO) at 37°C with 5% CO_2_. Human bronchial epithelial Calu-3 cells were cultured in DMEM-nutrient mixture F-12 Hams (1:1) supplemented with 1% MEM nonessential amino acids solution (GIBCO), 1% GlutaMAX (GIBCO), and 10% FBS (GIBCO) at 37°C with 5% CO_2_. Calu-3 cells were seeded at a density of 5 × 10^5^ cells/cm^2^ on semipermeable membrane inserts (0.6 cm^2^; Millicell-PCF; Millipore). After 3 to 4 weeks of culture for differentiation, cells with transepithelial electrical resistance (TEER) values over 600 V/cm^2^ were used for HBoV infection.

### Transfection.

Plasmids (2 μg) were transfected into cells plated on 60-mm dishes with Lipofectamine 2000 reagent (Invitrogen, Life Technologies) according to the manufacturer's instructions.

### Virus production and purification.

The infectious clone pHBoV1-WH (10 μg) was transfected into HEK293T cells seeded on 100-mm plates with Lipofectamine 2000. Forty-eight hours posttransfection, the cells were collected and lysed by three rounds of freezing and thawing. The cell lysate was then spun at 10,000 rpm for 30 min after Benzonase (Sigma) treatment for 30 min at 37°C. The supernatant was collected and further purified through discontinuous step gradients of iodixanol, prepared using a 60% (wt/vol) sterile solution of OptiPrep (Axis-Shield) as described previously (44). Viral DNA was extracted using a QIAamp blood minikit (Qiagen) and quantified using quantitative PCR as described previously (21).

### Virus infection.

Differentiated Calu-3 cells in Millicell inserts were incubated with HBoV1 virus purified from transfected HEK293T cells at a multiplicity of infection (MOI) of 100 g/cell at 37°C for 2 h, followed by three washes with phosphate-buffered saline (PBS). The cells were then cultured for HBoV RNA analysis.

### Plasmid construction. (i) HBoV1 infectious clone construction.

The HBoV genome was prepared from the feces of a 2-year-old infant using a viral DNA extraction kit (Qiagen) according to the manufacturer's instructions. The nearly full-length genome (lacking the left-end hairpin [LEH] and right-end hairpin [REH] sequences) was amplified using primers (XhoI-F, 5′-GCGCTCGAGCCGGCAGACATATTGGATTC-3′; XbaI-R, 5′-GCGTCTAGATGTACAACAACAACACATTAAAAGATATAGAG-3′). LEH and REH were synthesized according to the reference sequence JQ923422. The full-length recombinant HBoV clone was ligated into the pBBSmal vector and designated pHBoV1-WH (21, 45).

### (ii) Construction of pHBoV1-WH mutants.

All of the nonstructural and structural protein knockout plasmids were constructed based on the recombinant HBoV clone pHBoV1-WH by single-nucleotide mutation that resulted in early translation termination of the open reading frame. NS1, NP1, VP1, and VP2 open reading frames were disrupted by mutating nt 542 from T to A, nt 2588 from G to A, nt 3205 from T to A, and nt 3540 from T to G, respectively (Fig. 3A).

### (iii) Inverted terminal repeat (ITR) mutants.

LEH truncation plasmids pHBoV1LEH-39, pHBoV1LEH-70, pHBoV1LEH-96, and pHBoV1LEH-140 were constructed by deleting the first 39, 70, 96, and 140 nt, respectively, of HBoV in pHBoV1-WH. Similarly, the right-end mutant plasmids pHBoV1REH-98 and pHBoV1REH-161 were constructed by truncating the last 98 and 161 nt of HBoV in pHBoV1LEH-140, which does not contain the LEH.

### (iv) Reporter constructs to test *cis* elements required for polyadenylation at (pA)d2.

The parent polyadenylation reporter plasmid pEGFP-C1(-SV40 PA) was generated by deleting the SV40 polyadenylation signal of pEGFP-C1. The (pA)d2 reporter plasmids pEGFP-(pA)d and pEGFP-(pA)d2 were constructed by inserting the HBoV sequences of nt 5121 to 5271 and nt 5121 to 5543, respectively, into pEGFP-C1(-SV40 PA). pEGFP-HS was constructed by replacing nt 5121 to 5355 with the heterologous sequences from the open reading frame of kanamycin spanning from nt 57 to nt 291 based on plasmid pEGFP-(pA)d2. pEGFP-sPA was generated by changing nt 5153 to 5201 with a synthesized polyadenylation signal (5′-AATAAAAGATCTTTATTTTCATTAGATCTGTGTGTTGGTTTTTTGTGTG-3′) based on plasmid pEGFP-(pA)d2. pEGFP-mPAS was constructed by replacing (pA)d hexanucleotide AAUAAA with CCTAGG based on pEGFP-(pA)d2.

### (v) Constructs to identify the DSE of polyadenylation at (pA)d site.

pHBoV1-WH(NheI-SmaI) was constructed by mutating nt 4918 to 4921 from CTCA to TAGC and nt 5350 to 5355 from ATGCAA to CCCGGG, resulting in NheI and SmaI restriction enzyme sites in pHBoV1-WH. pHBoV1mDSE1, pHBoV1mDSE2, pHBoV1mDSE3, and pHBoV1mDSE4 were constructed by mutating nt 5159 to 5196, nt 5177 to 5196, nt 5159 to 5177, and nt 5159 to 5201 to 5′-CACTGACTGTCAGTCTGAGTCAGAGTCACTGACTCAGC-3′, 5′-TCAGAGTCACTGACTCAGC-3′, 5′-CACTGACTGTCAGTCTGAG-3′, and synthetic polyadenylation signal (5′-AGATCTTTATTTTCATTAGATCTGTGTGTTGGTTTTTTGTGTG-3′), respectively, in pHBoV1-WH(NheI-SmaI) (46, 47).

### (vi) Constructs to analyze effect of the distance between (pA)d and (pA)d2 on polyadenylation at (pA)d2 site.

pHBoV1Del1 and pHBoV1Del2 plasmids were made by deleting nt 5223 to nt 5356 and nt 5197 to nt 5356 on pHBoV1-WH(NheI-SmaI) to shorten the distance between (pA)d and (pA)d2. pHBoV1mut1 was mutated from nt 5223 to nt 5356 with a kanamycin open reading frame from nt 4 to nt 117. pHBoV1HS1 and pHBoV1HS2 were constructed by replacing the sequences from nt 5223 to nt 5356 with a kanamycin open reading frame from nt 4 to nt 217 and nt 4 to nt 617, respectively.

### (vii) Construction of NS1, NP1, and VP2 expression plasmids.

NS1 expression plasmid pcDNA-NS1 was constructed by inserting the NS1 ORF into pcDNA3.1 vector with a C-terminal hemagglutinin (HA) tag. NP1 ORF, VP2 ORF, and downstream sequences were inserted into pXJ40-FLAG to generate NP1 (pXJ40-NP1) and VP2 expression plasmids [pXJ40-VP2(pA)d and pXJ40-VP2(pA)d2]. The plasmid pXJ40-VP2(pA)d contains only the (pA)d sequence. The plasmid pXJ40-VP2(pA)d2 contains both (pA)d and (pA)d2 sequences (Fig. 9A).

### Southern blot analysis.

Isolation of low-molecular-weight (Hirt) DNA and Southern analysis were performed as previously described (29, 48) with digoxigenin (DIG)-labeled DNA probes (Roche).

### RNA isolation.

Total RNA from transfected cells was harvested using TRIzol reagent (Ambion) according to the manufacturer's instructions.

### 3′ RACE.

Reverse transcription was performed with total RNA from transfected or infected cells with oligo(dT) primers (5′-GCTATCATCACAATGGACTTTTTTTTTTTTTTTTTTV-3′). PCR amplification was performed using an adaptor primer (5′-GCTATCATCACAATGGAC-3′) and a gene-specific primer (GSP1, 5′-CCAACTAACCTAGAATACAAACTTC-3′). The second round of PCR was performed with the adaptor primer and another gene-specific primer (GSP2, 5′-CAGATTTCCTATCACCAGAG-3′), and the PCR products were gel purified with the gel extraction kit (Omega) according to the manufacturer's protocol and then sequenced.

### RNase H cleavage and Northern blotting.

Total RNA (20 μg) was combined with 100 pmol oligo(dT) (5′-TTTTTTTTTTTTTTTTTTTT-3′) and a gene-specific primer (5′-CATCCATATGTCCCCCACTA-3′). The mixture was incubated at 65°C for 5 min, followed by slow cooling to room temperature. RNase H buffer and enzyme (5 U) were added and samples were incubated at 37°C for 1 h. RNase H-treated samples were ethanol precipitated for at least 30 min at −20°C and then centrifuged. The RNA pellet was dissolved and run on 1.5% agarose gel containing 2.2 M formaldehyde for 12 h at 28 V. RNA was transferred to Hybond-N^+^ membrane by semidry transferring and then cross-linked using UV. Probe detection was performed by using the DIG luminescence detection kit II (Roche) according to the manufacturer's protocol. Signals were detected with the ChemiDoc MP imaging system (Bio-Rad).

### Western blotting.

Cell lysates were prepared 48 h after transfection and were separated by SDS–12% PAGE, followed by transfer to nitrocellulose. Protein detection was carried out using standard protocols with anti-HA antibody (66006; Proteintech), anti-GFP (66002; Proteintech), anti-FLAG (F1804; Sigma), and anti-actin (sc-47778; Santa Cruz Biotechnology). Luminescent signals were detected with the ChemiDoc MP imaging system (Bio-Rad).

### Statistical analysis.

3′ RACE was repeated at least three times. The ratio of polyadenylation at (pA)d2 to (pA)d was quantified. The averages and standard deviations are presented.

## References

[1] Allander T, Tammi MT, Eriksson M, Bjerkner A, Tiveljung-Lindell A, Andersson B 2005 Cloning of a human parvovirus by molecular screening of respiratory tract samples. Proc Natl Acad Sci U S A 102:12891–12896. doi:10.1073/pnas.0504666102. 16118271 PMC1200281

[2] Cotmore SF, Agbandje-McKenna M, Chiorini JA, Mukha DV, Pintel DJ, Qiu J, Soderlund-Venermo M, Tattersall P, Tijssen P, Gatherer D, Davison AJ 2014 The family Parvoviridae. Arch Virol 159:1239–1247. doi:10.1007/s00705-013-1914-1. 24212889 PMC4013247

[3] Risku M, Katka M, Lappalainen S, Rasanen S, Vesikari T 2012 Human bocavirus types 1, 2 and 3 in acute gastroenteritis of childhood. Acta Paediatr 101:e405–e410. doi:10.1111/j.1651-2227.2012.02727.x. 22568605 PMC7159669

[4] Koseki N, Teramoto S, Kaiho M, Gomi-Endo R, Yoshioka M, Takahashi Y, Nakayama T, Sawada H, Konno M, Ushijima H, Kikuta H, Ariga T, Ishiguro N 2012 Detection of human bocaviruses 1 to 4 from nasopharyngeal swab samples collected from patients with respiratory tract infections. J Clin Microbiol 50:2118–2121. doi:10.1128/JCM.00098-12. 22442328 PMC3372153

[5] Khamrin P, Thongprachum A, Shimizu H, Okitsu S, Mizuguchi M, Hayakawa S, Maneekarn N, Ushijima H 2012 Detection of human bocavirus 1 and 2 from children with acute gastroenteritis in Japan. J Med Virol 84:901–905. doi:10.1002/jmv.23274. 22499013

[6] Khamrin P, Malasao R, Chaimongkol N, Ukarapol N, Kongsricharoern T, Okitsu S, Hayakawa S, Ushijima H, Maneekarn N 2012 Circulating of human bocavirus 1, 2, 3, and 4 in pediatric patients with acute gastroenteritis in Thailand. Infect Genet Evol 12:565–569. doi:10.1016/j.meegid.2012.01.025. 22333841

[7] Guo L, Wang Y, Zhou H, Wu C, Song J, Li J, Paranhos-Baccala G, Vernet G, Wang J, Hung T 2012 Differential seroprevalence of human bocavirus species 1-4 in Beijing, China. PLoS One 7:e39644. doi:10.1371/journal.pone.0039644. 22761854 PMC3382199

[8] Cashman O, O'Shea H 2012 Detection of human bocaviruses 1, 2 and 3 in Irish children presenting with gastroenteritis. Arch Virol 157:1767–1773. doi:10.1007/s00705-012-1343-6. 22614812

[9] Wang Y, Gonzalez R, Zhou H, Li J, Li Y, Paranhos-Baccala G, Vernet G, Guo L, Wang J 2011 Detection of human bocavirus 3 in China. Eur J Clin Microbiol Infect Dis 30:799–805. doi:10.1007/s10096-011-1159-4. 21286929

[10] Kantola K, Hedman L, Arthur J, Alibeto A, Delwart E, Jartti T, Ruuskanen O, Hedman K, Soderlund-Venermo M 2011 Seroepidemiology of human bocaviruses 1-4. J Infect Dis 204:1403–1412. doi:10.1093/infdis/jir525. 21921203 PMC3988444

[11] Santos N, Peret TC, Humphrey CD, Albuquerque MC, Silva RC, Benati FJ, Lu X, Erdman DD 2010 Human bocavirus species 2 and 3 in Brazil. J Clin Virol 48:127–130. doi:10.1016/j.jcv.2010.03.014. 20382557

[12] Han TH, Kim CH, Park SH, Kim EJ, Chung JY, Hwang ES 2009 Detection of human bocavirus-2 in children with acute gastroenteritis in South Korea. Arch Virol 154:1923–1927. doi:10.1007/s00705-009-0533-3. 19862470

[13] Arthur JL, Higgins GD, Davidson GP, Givney RC, Ratcliff RM 2009 A novel bocavirus associated with acute gastroenteritis in Australian children. PLoS Pathog 5:e1000391. doi:10.1371/journal.ppat.1000391. 19381259 PMC2663820

[14] Maggi F, Andreoli E, Pifferi M, Meschi S, Rocchi J, Bendinelli M 2007 Human bocavirus in Italian patients with respiratory diseases. J Clin Virol 38:321–325. doi:10.1016/j.jcv.2007.01.008. 17336143

[15] Weissbrich B, Neske F, Schubert J, Tollmann F, Blath K, Blessing K, Kreth HW 2006 Frequent detection of bocavirus DNA in German children with respiratory tract infections. BMC Infect Dis 6:109. doi:10.1186/1471-2334-6-109. 16834781 PMC1550408

[16] Sloots TP, McErlean P, Speicher DJ, Arden KE, Nissen MD, Mackay IM 2006 Evidence of human coronavirus HKU1 and human bocavirus in Australian children. J Clin Virol 35:99–102. doi:10.1016/j.jcv.2005.09.008. 16257260 PMC7108338

[17] Manning A, Russell V, Eastick K, Leadbetter GH, Hallam N, Templeton K, Simmonds P 2006 Epidemiological profile and clinical associations of human bocavirus and other human parvoviruses. J Infect Dis 194:1283–1290. doi:10.1086/508219. 17041855 PMC7199845

[18] Foulongne V, Olejnik Y, Perez V, Elaerts S, Rodiere M, Segondy M 2006 Human bocavirus in French children. Emerg Infect Dis 12:1251–1253. doi:10.3201/eid1708.060213.. 16965707 PMC3291226

[19] Choi EH, Lee HJ, Kim SJ, Eun BW, Kim NH, Lee JA, Lee JH, Song EK, Kim SH, Park JY, Sung JY 2006 The association of newly identified respiratory viruses with lower respiratory tract infections in Korean children, 2000-2005. Clin Infect Dis 43:585–592. doi:10.1086/506350. 16886150 PMC7107986

[20] Fry AM, Lu X, Chittaganpitch M, Peret T, Fischer J, Dowell SF, Anderson LJ, Erdman D, Olsen SJ 2007 Human bocavirus: a novel parvovirus epidemiologically associated with pneumonia requiring hospitalization in Thailand. J Infect Dis 195:1038–1045. doi:10.1086/512163. 17330795 PMC7109861

[21] Huang Q, Deng X, Yan Z, Cheng F, Luo Y, Shen W, Lei-Butters DC, Chen AY, Li Y, Tang L, Soderlund-Venermo M, Engelhardt JF, Qiu J 2012 Establishment of a reverse genetics system for studying human bocavirus in human airway epithelia. PLoS Pathog 8:e1002899. doi:10.1371/journal.ppat.1002899. 22956907 PMC3431310

[22] Chen AY, Cheng F, Lou S, Luo Y, Liu Z, Delwart E, Pintel D, Qiu J 2010 Characterization of the gene expression profile of human bocavirus. Virology 403:145–154. doi:10.1016/j.virol.2010.04.014. 20457462 PMC2879452

[23] Tewary SK, Zhao H, Shen W, Qiu J, Tang L 2013 Structure of the NS1 protein N-terminal origin recognition/nickase domain from the emerging human bocavirus. J Virol 87:11487–11493. doi:10.1128/JVI.01770-13. 23966383 PMC3807368

[24] Sukhu L, Fasina O, Burger L, Rai A, Qiu J, Pintel DJ 2013 Characterization of the nonstructural proteins of the bocavirus minute virus of canines. J Virol 87:1098–1104. doi:10.1128/JVI.02627-12. 23135724 PMC3554049

[25] Fasina OO, Dong Y, Pintel DJ 2016 NP1 protein of the bocaparvovirus minute virus of canines controls access to the viral capsid genes via its role in RNA processing. J Virol 90:1718–1728. doi:10.1128/JVI.02618-15. PMC4733993 26637456

[26] Qu XW, Liu WP, Qi ZY, Duan ZJ, Zheng LS, Kuang ZZ, Zhang WJ, Hou YD 2008 Phospholipase A2-like activity of human bocavirus VP1 unique region. Biochem Biophys Res Commun 365:158–163. doi:10.1016/j.bbrc.2007.10.164. 17981142

[27] Filippone C, Zhi N, Wong S, Lu J, Kajigaya S, Gallinella G, Kakkola L, Soderlund-Venermo M, Young NS, Brown KE 2008 VP1u phospholipase activity is critical for infectivity of full-length parvovirus B19 genomic clones. Virology 374:444–452. doi:10.1016/j.virol.2008.01.002. 18252260 PMC4283219

[28] Dijkman R, Koekkoek SM, Molenkamp R, Schildgen O, van der Hoek L 2009 Human bocavirus can be cultured in differentiated human airway epithelial cells. J Virol 83:7739–7748. doi:10.1128/JVI.00614-09. 19474096 PMC2708629

[29] Guan W, Cheng F, Yoto Y, Kleiboeker S, Wong S, Zhi N, Pintel DJ, Qiu J 2008 Block to the production of full-length B19 virus transcripts by internal polyadenylation is overcome by replication of the viral genome. J Virol 82:9951–9963. doi:10.1128/JVI.01162-08. 18684834 PMC2566258

[30] Huang Q, Deng X, Best SM, Bloom ME, Li Y, Qiu J 2012 Internal polyadenylation of parvoviral precursor mRNA limits progeny virus production. Virology 426:167–177. doi:10.1016/j.virol.2012.01.031. 22361476 PMC3294060

[31] Qiu J, Cheng F, Johnson FB, Pintel D 2007 The transcription profile of the bocavirus bovine parvovirus is unlike those of previously characterized parvoviruses. J Virol 81:12080–12085. doi:10.1128/JVI.00815-07. 17715221 PMC2168810

[32] Sun Y, Chen AY, Cheng F, Guan W, Johnson FB, Qiu J 2009 Molecular characterization of infectious clones of the minute virus of canines reveals unique features of bocaviruses. J Virol 83:3956–3967. doi:10.1128/JVI.02569-08. 19211770 PMC2663281

[33] Zou W, Cheng F, Shen W, Engelhardt JF, Yan Z, Qiu J 2016 Nonstructural protein NP1 of human bocavirus 1 plays a critical role in the expression of viral capsid proteins. J Virol 90:4658–4669. doi:10.1128/JVI.02964-15. 26912614 PMC4836317

[34] Liu JM, Green SW, Shimada T, Young NS 1992 A block in full-length transcript maturation in cells nonpermissive for B19 parvovirus. J Virol 66:4686–4692. 1385833 10.1128/jvi.66.8.4686-4692.1992 PMC241293

[35] Yoto Y, Qiu J, Pintel DJ 2006 Identification and characterization of two internal cleavage and polyadenylation sites of parvovirus B19 RNA. J Virol 80:1604–1609. doi:10.1128/JVI.80.3.1604-1609.2006. 16415037 PMC1346959

[36] Shen W, Deng X, Zou W, Cheng F, Engelhardt JF, Yan Z, Qiu J 2015 Identification and functional analysis of novel nonstructural proteins of human bocavirus 1. J Virol 89:10097–10109. doi:10.1128/JVI.01374-15. 26223640 PMC4577888

[37] Tian B, Manley JL 2013 Alternative cleavage and polyadenylation: the long and short of it. Trends Biochem Sci 38:312–320. doi:10.1016/j.tibs.2013.03.005. 23632313 PMC3800139

[38] Sandberg R, Neilson JR, Sarma A, Sharp PA, Burge CB 2008 Proliferating cells express mRNAs with shortened 3′ untranslated regions and fewer microRNA target sites. Science 320:1643–1647. doi:10.1126/science.1155390. 18566288 PMC2587246

[39] Shen W, Deng X, Zou W, Engelhardt JF, Yan Z, Qiu J 2016 Analysis of the cis and trans requirements for DNA replication at the right end hairpin of the human bocavirus 1 genome. J Virol 90:7761–7777. doi:10.1128/JVI.00708-16. 27334591 PMC4988151

[40] Babkin IV, Tyumentsev AI, Tikunov AY, Zhirakovskaia EV, Netesov SV, Tikunova NV 2015 A study of the human bocavirus replicative genome structures. Virus Res 195:196–202. doi:10.1016/j.virusres.2014.10.019. 25449911

[41] Zhao H, Zhao L, Sun Y, Qian Y, Liu L, Jia L, Zhang Y, Dong H 2012 Detection of a bocavirus circular genome in fecal specimens from children with acute diarrhea in Beijing, China. PLoS One 7:e48980. doi:10.1371/journal.pone.0048980. 23133667 PMC3487788

[42] Brinton MA, Basu M 2015 Functions of the 3′ and 5′ genome RNA regions of members of the genus Flavivirus. Virus Res 206:108–119. doi:10.1016/j.virusres.2015.02.006. 25683510 PMC4540327

[43] Pijlman GP, Funk A, Kondratieva N, Leung J, Torres S, van der Aa L, Liu WJ, Palmenberg AC, Shi PY, Hall RA, Khromykh AA 2008 A highly structured, nuclease-resistant, noncoding RNA produced by flaviviruses is required for pathogenicity. Cell Host Microbe 4:579–591. doi:10.1016/j.chom.2008.10.007. 19064258

[44] Zolotukhin S, Byrne BJ, Mason E, Zolotukhin I, Potter M, Chesnut K, Summerford C, Samulski RJ, Muzyczka N 1999 Recombinant adeno-associated virus purification using novel methods improves infectious titer and yield. Gene Ther 6:973–985. doi:10.1038/sj.gt.3300938. 10455399

[45] Zhi N, Mills IP, Lu J, Wong S, Filippone C, Brown KE 2006 Molecular and functional analyses of a human parvovirus B19 infectious clone demonstrates essential roles for NS1, VP1, and the 11-kilodalton protein in virus replication and infectivity. J Virol 80:5941–5950. doi:10.1128/JVI.02430-05. 16731932 PMC1472615

[46] Levitt N, Briggs D, Gil A, Proudfoot NJ 1989 Definition of an efficient synthetic poly(A) site. Genes Dev 3:1019–1025. doi:10.1101/gad.3.7.1019. 2570734

[47] Guan W, Huang Q, Cheng F, Qiu J 2011 Internal polyadenylation of the parvovirus B19 precursor mRNA is regulated by alternative splicing. J Biol Chem 286:24793–24805. doi:10.1074/jbc.M111.227439. 21622561 PMC3137055

[48] Guan W, Wong S, Zhi N, Qiu J 2009 The genome of human parvovirus b19 can replicate in nonpermissive cells with the help of adenovirus genes and produces infectious virus. J Virol 83:9541–9553. doi:10.1128/JVI.00702-09. 19587029 PMC2738243

